# Development of monoclonal antibodies and serological assays including indirect ELISA and fluorescent microsphere immunoassays for diagnosis of porcine deltacoronavirus

**DOI:** 10.1186/s12917-016-0716-6

**Published:** 2016-06-08

**Authors:** Faten Okda, Steven Lawson, Xiaodong Liu, Aaron Singrey, Travis Clement, Kyle Hain, Julie Nelson, Jane Christopher-Hennings, Eric A. Nelson

**Affiliations:** Veterinary & Biomedical Sciences Department, South Dakota State University, Brookings, SD USA; National Research Center, Giza, Egypt

**Keywords:** Porcine deltacoronavirus (PDCoV), Monoclonal antibodies, Serology, ELISA, Fluorescent microsphere immunoassay (FMIA)

## Abstract

**Background:**

A novel porcine deltacoronavirus (PDCoV), also known as porcine coronavirus HKU15, was reported in China in 2012 and identified in the U.S. in early 2014. Since then, PDCoV has been identified in a number of U.S. states and linked with clinical disease including acute diarrhea and vomiting in the absence of other identifiable pathogens. Since PDCoV was just recently linked with clinical disease, few specific antibody-based reagents were available to assist in diagnosis of PDCoV and limited serological capabilities were available to detect an antibody response to this virus. Therefore, the overall objective of this project was to develop and validate selected diagnostic reagents and assays for PDCoV antigen and antibody detection.

**Results:**

The nucleoprotein of PDCoV was expressed as a recombinant protein and purified for use as an antigen to immunize mice for polyclonal, hyperimmune sera and monoclonal antibody (mAb) production. The resulting mAbs were evaluated for use in fluorescent antibody staining methods to detect PDCoV infected cells following virus isolation attempts and for immunohistochemistry staining of intestinal tissues of infected pigs. The same antigen was used to develop serological tests to detect the antibody response to PDCoV in pigs following infection. Serum samples from swine herds with recent documentation of PDCoV infection and samples from expected naïve herds were used for initial assay optimization. The tests were optimized in a checkerboard fashion to reduce signal to noise ratios using samples of known status. Statistical analysis was performed to establish assay cutoff values and assess diagnostic sensitivities and specificities. At least 629 known negative serum samples and 311 known positive samples were evaluated for each assay. The enzyme linked immunosorbent assay (ELISA) showed diagnostic sensitivity (DSe) of 96.1 % and diagnostic specificity (DSp) of 96.2 %. The fluorescent microsphere immunoassay (FMIA) showed a DSe of 95.8 % and DSp of 98.1 %. Both ELISA and FMIA detected seroconversion of challenged pigs between 8–14 days post-infection (DPI). An indirect fluorescent antibody (IFA) test was also developed using cell culture adapted PDCoV for comparative purposes.

**Conclusion:**

These new, specific reagents and serological assays will allow for improved diagnosis of PDCoV. Since many aspects of PDCoV infection and transmission are still not fully understood, the reagents and assays developed in this project should provide valuable tools to help understand this disease and to aid in the control and surveillance of porcine deltacoronavirus outbreaks.

## Background

Coronaviruses are enveloped, positive sense RNA viruses divided among several genera, including *Alphacoronavirus*, *Betacoronavirus*, *Gammacoronavirus* and the recently described genus *Deltacoronavirus*. A novel porcine deltacoronavirus (PDCoV) was reported in China in 2012 and designated HKU15 [1]. Other important porcine coronaviruses include porcine epidemic diarrhea virus (PEDV), transmissible gastroenteritis virus (TGEV) and porcine respiratory coronavirus (PRCV); and are members of the genus *Alphacoronavirus* [2]. In February 2014, the Ohio Department of Agriculture announced the identification of PDCoV in swine feces at five farms in Ohio and associated with enteric disease similar to PEDV in the U.S. [3]. Since then, PDCoV has been identified in numerous U.S. states and Canada, linked with apparent clinical disease including acute diarrhea and vomiting in the absence of other identifiable pathogens. According to field observations in the U.S., PDCoV infections cause less severe clinical disease than PEDV, but analysis of the field data is complicated since co-infections with PEDV or other pathogens are common. PDCoV is currently diagnosed by real time PCR and clinical symptoms [1, 4].

The severity of disease in both gnotobiotic and conventional piglets has further defined the pathogenicity and pathogenesis of the virus [5–7]. PDCoV causes diarrhea and vomiting in all age groups and mortality in nursing pigs but the mortality rates are less than that shown in cases of PEDV. Previously, there was little information about deltacoronavirus infections in pigs and only one surveillance study from Hong Kong reported its detection in pigs prior to its emergence in the U.S. The virus had not been reported to be associated with clinical disease in China. The newly emergent strain found on the Ohio farms, PorCoV HKU15 OH 1987, is closely related to the 2 strains from China, but it is unknown how this virus was introduced into the US [3].

Recently, Jung et al. [7] developed in-situ hybridization and immunofluorescence staining techniques to demonstrate the areas of PDCoV replication in tissues of infected pigs. The OH-FD22 and OH-FD100 PDCoV strains were confirmed as causing an acute infection through the entire intestine, but primarily the jejunum and ileum, and clinically lead to severe diarrhea and vomiting. Clinical signs and pathological features of PDCoV-infected pigs resemble those of PEDV and TGEV infections. Effective differential diagnosis between PDCoV, PEDV, and TGEV is important to control the diseases.

Polymerase chain reaction (PCR) assays were quickly developed for the detection of PDCoV infections following the initial U.S. identification in 2014 but available serological assays are limited. Thachil et al. [8] developed an indirect anti-PDCoV IgG enzyme-linked immunosorbent assay (ELISA) based on the S1 portion of the spike protein. Although this assay was shown to be a highly sensitive (91 %) and specific test (95 %), there is need for other ELISAs utilizing alternative antigen targets, such as the nucleoprotein of PDCoV, to serve as primary serological surveillance or confirmatory assays. As noted in Thachil’s research, several serum samples collected in 2010 were found positive for PDCoV antibody by their ELISA, but all those collected in 2011 and 2012 tested negative by that assay. This finding is interesting since PDCoV was not thought to be circulating in North America prior to late 2013 [7, 9]. Therefore, availability of several serological assay formats targeting different viral antigens can be valuable as confirmatory assays in the investigation of unexpected laboratory findings.

Since no specific antibody-based reagents were available to assist in diagnosis of PDCoV, one purpose of the current study was to develop readily available reagents for detection of PDCoV antigen in diagnostic tests, such as virus isolation, immunohistochemistry and fluorescent antibody techniques. Serological tests for the detection of antibody responses to PDCoV were also very limited. Therefore, another objective of this study was to develop and optimize several serological assays including an indirect ELISA, a fluorescent microsphere immunoassay (FMIA), and an indirect fluorescent antibody (IFA) test.

Both specific antibody-based reagents and serological tests are essential for the further study and control of PDCoV and the differentiation of PDCoV infection from other related diseases such as PEDV or TGEV. The tools developed during the course of this study can be applied to many ongoing and future studies to better understand and control PDCoV.

## Methods

### Serum samples

Samples used for optimization and validation of the PDCoV ELISA and FMIA assays included samples from a large PDCoV challenge study associated with National Pork Board (NPB) research project 14–182. These samples and samples from another group of 30 pigs which were collected near the time of initial field exposure to PDCoV and 28 days later were used in a time course study.

For further validation and assessment of diagnostic sensitivity (DSe) and diagnostic specificity (DSp), samples of known PDCoV serostatus were used (*n* = 940). The expected positive samples were submitted field serum samples (*n* = 311) from herds previously testing PDCoV positive by PCR at least 3 weeks prior to sample collection. Expected negative samples included archived experimental serum collected prior to 2009 (*n* = 108) and field samples from high-health herds with no known history of PDCoV exposure (*n* = 521). The total number of expected negative samples was 629.

### Viruses and cells

Swine testicle (ST) cells were cultured in Eagle’s minimum essential medium (MEM; Gibco BRL Life Technologies) supplemented with 10 % fetal bovine serum (FBS), and antibiotics (100 units/ml penicillin and 20 g/ml streptomycin). Cells were maintained at 37 °C in a humidified 5 % CO_2_ incubator. Cell culture adapted PDCoV was provided by the National Veterinary Services Laboratories, designated porcine coronavirus HKU15 strain Michigan/8977/2014 (GenBank accession KM012168). PDCoV was propagated on ST cells utilizing 0.8 μg/ml trypsin (TPCK-treated, bovine derived (Sigma, St. Louis, MO)) in the inoculation and maintenance media. Virus infected cells were harvested 24–48 h after inoculation, when significant cytopathic effect (CPE) was noted.

### Antigen production

The antigen used for the FMIA and indirect ELISA validation was a recombinantly expressed, full length, PDCoV nucleoprotein (NP). RNA isolated from semi-purified cultured virus corresponding to the PDCoV-NP was amplified by reverse transcriptase polymerase chain reaction (RT-PCR) and resulting DNA cloned into the pET-28a protein expression vector (Novagen, Madison, WI). Primers used for amplification of the nucleocapsid region are described:

PDCoV-NP fwd (5′-CGCGGATCCATGGCCGCACCAGTAGTC - 3′);

PDCoV-NP rev (5′-CACACTCGAGCGCGCTGCTGATTCCTGCTT- 3′).

The NP gene was prokaryotically expressed as an insoluble 41 kDa, 6x polyhistidine-tagged, fusion protein then purified according to previously described methods [10]. Purified protein was analyzed by sodium dodecyl sulfate- polyacrylamide gel electrophoresis (SDS-PAGE) to determine purity and linear integrity. The expressed PDCoV NP was recognized in Western blotting by convalescent serum and two separate monoclonal antibodies developed in our laboratory were used to confirm the specificity of the proteins.

### Refolding of the PDCoV NP purified protein

The purified protein was refolded by first solubilizing the recombinant protein expressed as insoluble inclusion bodies in *E. coli.* Briefly, the protein was solubilized in 50 mM 3-(Cyclohexylamino)-1-propanesulfonic acid (CAPS buffer, pH 11.0) containing 1.0 % N-lauroylsarcosine and 1.0 mM DTT. The protein was then dialyzed overnight at 4 °C in 20 mM Tris–HCl pH 8.5 containing 0.1 mM DTT to encourage correct disulfide bond formation and subsequent refolding of the protein. A second dialysis step was done in phosphate buffered saline (PBS) to remove excess reducing agent. Testing of the re-folded NP-based ELISA and FMIA began with checkerboard titrations of both the antigen and PDCoV convalescent sera to determine optimum concentrations of each. Depending on the calculation of signal to noise ratios, optimum concentration of NP antigen was identified for coating of the ELISA plates and coupling FMIA beads. We also identified optimum test serum dilutions and blocking agents.

### Development and diagnostic application of rabbit antisera and monoclonal antibodies

Rabbits and mice were immunized with selected recombinant PDCoV proteins for production of hyperimmune antisera and monoclonal antibodies (mAbs) as previously described [11–13]. Immunoglobulin isotyping of the resulting mAbs was performed using a commercial lateral flow assay (Serotec, Raleigh, NC).

### Indirect fluorescent antibody assay

An IFA assay was developed for reference purposes using pig serum of known serostatus. ST cells were grown in cultures for 2 to 3 days to 80 % confluence on 96-well plates. Odd numbered lanes were infected with PDCoV (approximately 1000 50 % tissue culture infective doses (TCID_50_)/ml) in MEM supplemented with 0.8 ug/ml TPCK-treated trypsin. The plates were incubated for 18 to 24 h. then fixed with 50 % (vol/vol) acetone/methanol for 20 min at −20 °C, air dried, and frozen with a desiccant at −20 °C until they were used. Serum dilutions of 1:20 and 1:40 were applied to infected and control wells of the IFA plates and incubated 1 h. After washing three times with 300 μl of phosphate buffered saline (PBS), 40 μl of fluorescein isothiocyanate (FITC)-labeled goat anti-swine immunoglobulin G (41.7 g/ml; KPL, West Chester, PA) was added to each well. The plates were incubated at 37 °C for 1 h and washed with PBS three times. The cells were examined for specific fluorescence with an inverted microscope and a UV light source (Nikon Eclipse TS100). Serum samples were considered positive if PDCoV specific fluorescence was observed at the 1:20 serum dilution.

### Antibody detection indirect ELISA

The refolded PDCoV NP antigen-based indirect ELISA was performed using methods previously described in Okda et al. [13]. Briefly, alternate wells of Immulon 1B, 96-well, microtiter plates (Thermo Labsystems, Franklin, MA) were coated for 1 h at 37 °C with 200 ng/well of purified, refolded PDCoV-NP antigen diluted in 15 mM sodium carbonate-35 mM sodium bicarbonate, antigen coating buffer (ACB) pH 9.6. Next, non-bound antigen was poured off and the plates washed 3X with PBS plus 0.05 % tween-20 (PBST), then the remaining free-binding sites were blocked with 200 μl of sample milk diluent ((SMD)-PBST plus 5 % nonfat dry milk) and incubated overnight at 4 °C. Test and control sera were diluted 1:50 in SMD, and 100 μl of the solution was added to each well of a washed plate. The plates were incubated for 1 h at 22 °C. Next, 100 μl of biotinylated, FC-specific, goat anti-swine detection antibody (Bethyl Laboratories, Montgomery, TX) was diluted 1:4000 in PBST and allowed to incubate at 22 °C for 1 h. Plates were washed 3X, then 100 μl of streptavidin-horseradish peroxidase conjugate (Pierce, Rockford, IL, diluted 1:4000) was added and incubated for 1 h at 22 °C, then washed and developed using TMB (Surmodics, Eden Prairie, MN). Colorimetric development was stopped using 2 N H_2_SO_4_, then OD’s were quantified spectrophotometrically at 450 nm with a ELx800 microplate reader (BioTek Instruments Inc., Winooski, VT). The raw OD’s were normalized and the corrected S/P values were calculated as follows: S/P = (OD of sample - OD of buffer)/(OD of positive control - OD of buffer).

The optimal dilution of the recombinant protein and secondary detection antibody was determined by a checkerboard titration that gave the highest signal to noise ratio. In addition, a single lot of pooled convalescent serum from PDCoV infected pigs was used to generate quality control standards that gave high, medium and low Sample to Positive (S/P) values. The negative, low and medium samples served as internal quality standards while the high standard served as a serum constant to mathematically calculate S/P values of individual unknowns. For the ELISA, a high positive S/P = 0.8–1.0; medium S/P = 0.6–0.8; low S/P = 0.4–0.6; and negative S/P < 0.2.

### Microsphere coupling and FMIA procedure

The coupling of purified, recombinant, refolded PDCoV-NP antigen to fluorescent microspheres was performed using a two-step, carbodiimide coupling reaction as previously described [14]. Prior to performing large scale coupling reactions for test validation, the optimization of the amount of antigen used was obtained by performing a checkerboard titration of antigen-coupled microspheres against a two-fold titration of swine serum. It was found that initiating a coupling reaction having 12.5ug of purified protein per 3.125 × 10^6^ microspheres was optimal in obtaining the highest signal-to-noise fluorescence ratio. The performance of the FMIA test was described in detail previously by Okda et al. [11]. In the initial optimization of the FMIA test, we performed two-fold serial dilutions of swine serum and concluded that a dilution of 1:50 provided the highest signal-to-noise ratio. For the generation of sample fluorescence, antigen-coupled microsphere/antibody complexes were analyzed through a dual-laser Bio-Rad Bio-Plex 200 instrument. The median fluorescent intensity (MFI) for 100 microspheres corresponding to each individual bead analyte was recorded for each well. All reported MFI measurements were normalized by calculating individual S/P values using the following formula: S/P = MFI of sample - MFI of buffer control/MFI of high positive control - MFI of buffer control. The buffer control equated to the background signal determined from the fluorescence measurement of antigen-coated beads. Lastly, a single lot of pooled convalescent serum from PDCoV infected pigs was used to generate quality control standards that gave high, medium and low S/P values. The negative, low and medium samples served as internal quality standards while the high standard served as a serum constant to mathematically calculate S/P values of individual unknowns. For the FMIA, a high positive S/P = 0.8–1.0; medium S/P = 0.6–0.8; low S/P = 0.4–0.6; and negative S/P < 0.2, is consistent with the data of the ELISA standards.

### Antibody capture efficacy comparison between refolded vs linear PDCoV-NP antigen

An antibody capture titration assay was employed to compare the efficacy of refolded vs. linear antigen to capture anti-PDCoV-NP specific antibody in swine serum. Wells of a 96-well microtiter plate were coated with 10-fold decreasing concentrations of either linearly expressed or refolded PDCoV-NP antigen in ACB, pH 9.6., then allowed to incubate for 1 h at 37 °C. Each well was then blocked with 200 μl of SMD and allowed to incubate overnight at 4 °C. The following day, the plates were washed 3X with 300 μl of PBST. Well characterized positive control sera having a “high” positive OD was diluted 1/50 in SMD, mixed, and 100 μl of the solution was added to each well. The ELISA was continued pursuant to the stated protocol and the OD was recorded at each titration point, then a logarithmic regression curve was generated. Relative capture efficiencies for each antigen-coated well was determined by analyzing the OD at each dilution point and position under the linear portion of the curve.

### Assay validation

(i) **Cutoff determination, DSe and DSp**. To accurately assess the DSe and DSp of the assays, the assays were validated using known seronegative and seropositive samples from distinct animal populations. The expected positive samples used were field serum samples submitted to the ADRDL from herds previously testing PDCoV positive by PCR. Expected negative samples included archived experimental serum collected prior to 2009 and field samples from high-health herds with no known history of PDCoV exposure. Receiver operating characteristic (ROC) analysis was performed using MedCalc version 11.1.1.0 (MedCalc software, Mariakerke, Belgium).
(ii) **Measurement of repeatability**. The repeatability of the FMIA and ELISA was assessed by running the same lot of internal quality control serum standards multiple times on the same plates and on different plates over time. The intra-assay repeatability was calculated using 36 replicates on a single plate and then repeated over a 3-day period for inter-assay repeatability assessment. Each assay was run in a single-plex format, and median fluorescence intensity values were expressed as percent coefficient of variation (CV) for repeated measurements. Percent CV was calculated using a method described earlier [15].

### Statistical analyses and measurement of testing agreement

Multiple comparison, inter-rater agreement (kappa measure of association) was calculated among all three tests (ELISA, FMIA and IFA) using IBM, SPSS version 20 software (SPSS Inc., Chicago, IL). The sample cohort used included a set of archived serum samples collected from PDCoV “positive testing” experimentally infected pigs over time and from archived experimental control uninfected PDCoV “negative testing” animals. The interpretation of kappa can be rated as follows: Kappa less than 0.0, “poor” agreement; between 0.0 and 0.20, “slight” agreement; between 0.21 and 0.40, “fair” agreement; between 0.41 and 0.60, “moderate” agreement; between 0.61 and 0.80, “substantial” agreement; and between 0.81 and 1.0, “almost perfect” agreement [16].

Validation of the tests was performed by ROC analysis using Medcalc statistical software. Correlations between the tests and scatterplots for seroconversion were performed using SPSS 20.

## Results

### Expression of recombinant full-length nucleoprotein

**Fig. 1:**
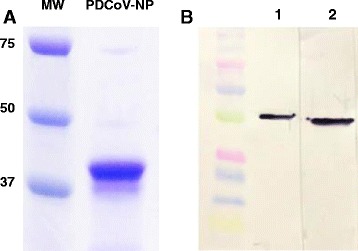
Purification and antigen specificity of PDCoV-NP antibody capture antigen. **a** Coomassie blue staining of *E. coli* expressed and purified PDCoV-NP antigen used to coat ELISA microtiter plates and FMIA microspheres. Molecular weight ladder MW and PDCoV-NP (41 kDa). **b** Western blot showing antigen specificity of recombinant, refolded PDCoV-NP/polyhistidine fusion protein probed with mAb SD-55-197 (Lane 1). Lane 2 was loaded with semi-purified, concentrated PDCoV strain HKU15/Michigan/8977/2014 then probed with mAb SD-55-197

The full-length NP of PDCoV was cloned and expressed in *E. coli* as a polyhistidine fusion protein. Antigen purity was then evaluated using SDS-PAGE in which the His-tagged recombinant NP migrated through the gel according to its predicted molecular mass of 41 kDa upon staining with Coomassie brilliant blue R250 (Fig. 1a). The recombinant protein was expressed in the form of insoluble inclusion bodies. It was purified by Nickel-NTA affinity column chromatography and yielded a calculated concentration of approximately 10 mg PDCoV-NP/liter of 2XYT medium and having a purity greater than 95 % as measured by the Lowry protein assay. The protein was subsequently refolded back to its soluble, conformational structure and its specificity was tested via Western blotting (Fig. 1b). The figure illustrates the migration pattern and antigen specificity of the refolded PDCoV-NP/polyhistidine fusion protein as compared to the adjacent lane loaded with semi-purified, concentrated PDCoV. Both the rPDCoV-NP and native virus are recognized with equal intensity by a PDCoV-NP-specific monoclonal antibody (SD55-197) developed in our laboratory. Also, the recombinant nucleocapsid protein is shown to have a higher molecular mass than the native virus nucleocapsid due to its dual amino and carboxy terminus polyhistidine tags.

**Fig. 2:**
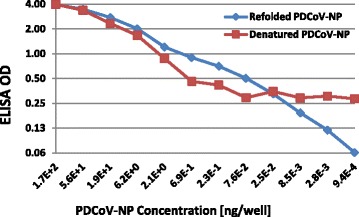
Antibody capture efficacy comparison between refolded vs linear PDCoV-NP. An ELISA antibody capture titration assay was employed to compare the ability of refolded vs. linear antigen to capture anti-PDCoV-NP specific antibody in swine serum. Wells of a 96-well microtiter plate were coated with decreasing concentrations of either linearly expressed or refolded PDCoV-NP antigen. Refolded antigen demonstrated greater dynamic range of the assay and capture efficacy of swine antibody

Experiments were conducted to assess the immunoreactivity of refolded vs non-refolded PDCoV-NP antigen used for both tests. Specifically, an antibody capture titration ELISA was employed to compare the ability of a refolded and non-refolded version of the antigen to capture antibodies within swine serum. The immunoreactivity of antigen was determined by end-point titration and relative absorbance values as we observed differences in immunoreactivity based upon the conformational state of antigen tested. Figure 2 demonstrates in a dose-dependent fashion, that as the concentration of each antigen coated well decreases, the refolded antigen imparts a greater degree of antibody capture efficacy of swine antibodies than the non-refolded version. Specifically, a 27 fold difference was calculated at the end of the linear portion of the curve indicating that the refolded protein maintained a marked enhancement of immunoreactivity resulting in a greater dynamic range of the assay.

### Fluorescent microsphere immunoassay and indirect ELISA development

**Fig. 3:**
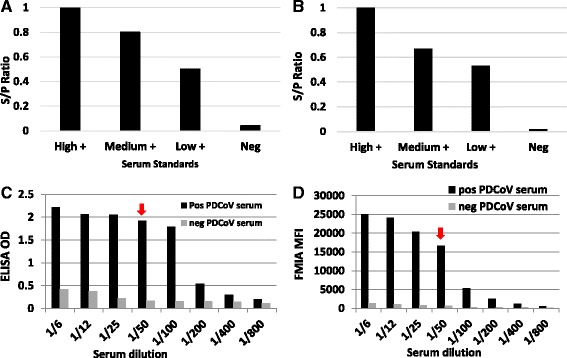
Production and reactivity of serological reference standards and internal quality control standards for both ELISA (**a**) & FMIA (**b**). Reference serum standards were titrated 2-fold in antigen coated wells at a fixed concentration [250 ng/well] in order to gauge a maximum signal-to-noise ratios for each assay **c** ELISA, and **d** FMIA. Arrows indicate the optimal serum dilution that resulted in the greatest positive to negative (P/N) ratio for the test
(ii) **Test optimization:** A series of coupling processes were performed using a two-fold titration of antigen to determine the optimum coupling concentration. A total of 3.125 x10^6^ beads, were incubated with various concentrations (100 μg, 50 μg, 25 μg, and 12.5 μg) of purified NP. Based upon the highest signal-to-noise ratio reflecting the detection of PDCoV-specific antibodies in standard serum, 12.5 μg per reaction was the optimal concentration for microsphere coupling. ELISA microtiter plates were coated with an optimized concentration of 200 ng antigen per well.

(i) **Establishment of control standards:** ELISA and FMIA test reference standards for PDCoV were developed for each of the respective prototype assays (Fig. 3a and b). A control high positive standard was established by pooling several lots of serum collected from convalescent, seropositive pigs, and used to mathematically calculate S/P values of each assay. The medium and low positive samples served as internal quality control standards. The negative standard was pooled from a set of known seronegative pigs from a herd with no known prior PDCoV infection that also tested negative by PDCoV IFA and virus neutralization.

Testing the re-folded, NP antigen-based ELISA and FMIA began with checkerboard titration of both antigen and PDCoV convalescent and naïve swine serum to determine the optimum signal-to-noise ratio of each test. We also identified optimum test serum dilutions and blocking agents. The optimal serum dilution of each assay was determined by diluting serum samples two-fold in their respective blocking/detergent buffer diluent. For both the FMIA and ELISA, a serum dilution of 1:50 was shown to produce an optimal signal-to-noise ratio (Fig. 3c and d). Testing field samples of known serological status was performed to gauge initial sensitivity of the assay. Our results showed a positive to negative sample ratio (P/N) of greater than 16-fold with ELISA and a P/N of greater than 40-fold with FMIA. The P/N is a relative measure of PDCoV antibody concentration between seropositive and seronegative samples. Having a diagnostic P/N of greater than 10 is highly desirable with any serological assay.

### Assessment of test repeatability

The intra-assay repeatability was calculated for 36 replicates on a single plate and then repeated over a 3-day period for inter-assay repeatability assessment. Internal control serum standards were used to determine the precision of each FMIA and ELISA. The inter-assay and intra-assay repeatability of each test demonstrated a coefficient of variation of less than 8.6 %. These results confirmed that the serological tests are highly repeatable in diagnostic applications.

### Validation methods and cutoff determination

**Fig. 4:**
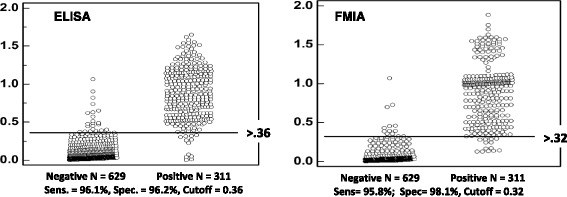
Receiver operator characteristic (ROC) validation and determination of diagnostic sensitivity (DSe) and specificity (DSp) of the PDCoV-NP ELISA and FMIA assays. DSe and DSp were calculated using serum samples from known PDCoV-infected and known PDCoV-uninfected populations. ROC analysis was performed using MedCalc version 11.1.1.0 (MedCalc software, Mariakerke, Belgium). In each panel, the dot plot on the left represents the negative population, and the plot on the right represents the positive population. The horizontal line bisecting the dot plots for each figure represents the tentative cutoff value that gives the optimal DSe and DSp

ROC analysis of both FMIA and ELISA was performed using MedCalc software to calculate an optimized cutoff value that maximizes the DSe and DSp of each assay. Using known seronegative and seropositive serum samples (*n* = 940), the expected positive samples used were submitted as field serum samples (*n* = 311) from herds previously testing PDCoV positive by PCR. Expected negative samples included archived experimental serum collected prior to 2009 (*n* = 108) and field samples from high-health herds with no known history of PDCoV exposure (*n* = 521). ROC analysis was performed and DSe and DSp were shown to be 96.1 and 96.2 % respectively for the ELISA; and 95.8 and 98.1 % respectively for the FMIA (Fig. 4). The similar cutoff values of both assays confirm the utility of these new diagnostic tests to aid in the control and surveillance of PDCoV outbreaks.

### Evaluation of the kinetic swine antibody response in serum

**Fig. 5:**
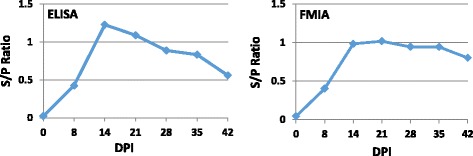
Serum antibody kinetic time course evaluation. Antibody kinetic responses were calculated for the ELISA and FMIA tests using serum collected weekly over six weeks from experimentally infected pigs. Serological responses detected by PDCoV ELISA and FMIA tests show seroconversion between 8 and 14 DPI

**Fig. 6:**
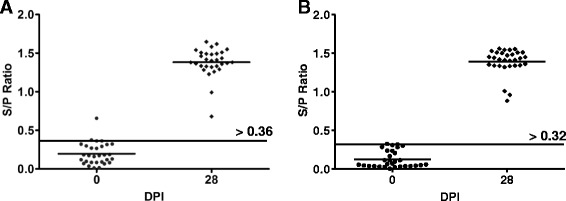
ELISA (**a**) and FMIA (**b**) results from a group of 30 piglets sampled near the time of initial PDCoV field exposure then 28 days later. Both assays show clear seroconversion to naturally circulating PDCoV. The horizontal line between both positive and negative testing samples shows the diagnostic cutoff value for each test previously determined by ROC analysis

Once validated, the assays were used to evaluate the kinetic antibody response over time using serum collected from experimentally infected pigs over weekly intervals. Serological responses detected by the PDCoV ELISA and FMIA following challenge show seroconversion between days 8 and 14 DPI (Fig. 5). Both assays demonstrate a similar dynamic range using the same serum samples and “high” positive standard from which the S/P values are calculated. However, the FMIA appears to detect a slightly higher level of antibody over a longer period of time at days 35 and 42 post infection. Additional testing of seroconversion included serum samples collected from a group of 30 piglets near the time of initial field exposure to PDCoV then 28 days later. Figure 6 shows clear seroconversion to the naturally circulating virus within the 28 day time-frame using the same diagnostic cutoff values previously determined by ROC analysis.

### Measurement of statistical testing agreement

Multiple comparison, inter-rater (kappa) agreement is a statistical measure of testing agreement, and was calculated among all three tests (ELISA, FMIA & IFA) using 629 positive testing and 311 negative testing serum samples. Statistical comparison calculated kappa values to be 0.940 between FMIA and IFA, 0.902 between ELISA and IFA, and 0.914 between ELISA and FMIA (Table 1). Because all three diagnostic platforms had kappa values above 0.81, it demonstrates that the tests are in “almost perfect” agreement with each other according to the interpretation of kappa by Landis et al. [16].

**Table 1:** Evaluation of statistical testing agreement among three serological testing platforms

	FMIA	Indirect ELISA	IFA
IFA	0.940	0.902	1
Indirect ELISA	0.914	1	0.902
FMIA	1	0.914	0.940
Number positive serum samples	311	311	315
Number negative serum samples	629	629	625
Total	940	940	940

Multiple comparison, inter-rater agreement (kappa association) were calculated among all three tests, IFA, indirect ELISA and FMIA. Kappa values shown represent a statistical measure of test agreement and were calculated using MedCalc version 11.1.1.0

### Cross reactivity

Serological cross reactivity testing was performed between PDCoV and other closely related swine coronaviruses. There was no cross reactivity among the 93 TGEV positive serum samples, 20 PRCV positive serum samples, 167 PEDV field positive serum samples and 84 PEDV experimentally positive serum samples tested via ELISA and FMIA (Table 2). The data show mean OD and MFI readings from ELISA and FMIA tests, respectively. The lack of cross reactivity between PDCoV and aforementioned alphacoronavirus species was also confirmed via western blotting using seropositive, convalescent sera from individual pigs (data not shown).

**Table 2:** Serological cross reactivity testing among related swine coronaviruses

	TGEV	PRCV	PEDV
ELISA Mean OD	0.097	0.132	0.074
FMIA Mean FI	0.024	0.016	0.032
Total No. serum samples tested	93	20	251

### Development of reagents for detection of PDCoV antigen in diagnostic tests

**Fig. 7:**
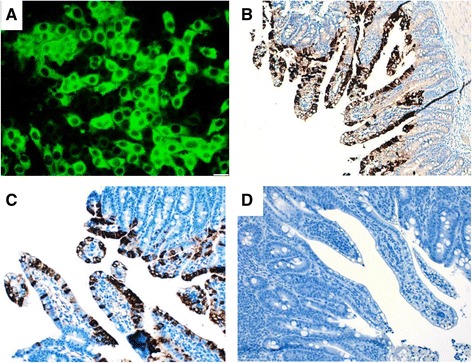
Development of reagents used for indirect fluorescent antibody (IFA) testing and immunohistochemical (IHC) staining of PDCoV infected cells. Indirect fluorescent antibody staining of PDCoV infected ST cells with PDCoV anti-nucleoprotein monoclonal antibody SD-55-24 [**a**, 100X magnification]. Immunohistochemistry staining of intestinal enterocytes with PDCoV anti-nucleoprotein rabbit polyclonal hyperimmune sera [**b**, 100X magnification]. Immunohistochemistry staining of intestinal enterocytes with PDCoV anti-nucleoprotein monoclonal antibody 55–197 [**c**, 100X magnification]. Uninfected control showing hematoxylin staining of luminal, intestinal brush-border cross section [**d**, 100X magnification]

Both denatured and refolded versions of the NP were used to immunize rabbits for hyperimmune serum and mice for monoclonal antibody production. Rabbit hyperimmune sera specifically recognize the NP and can be used in indirect fluorescent antibody staining at dilutions of 1:1000 to 1:5000. In addition, the polyclonal antisera was used successfully in immunohistochemical staining procedures for the detection of PDCoV antigen in intestinal tissues (Fig. 7b). The resulting monoclonal antibodies all recognized native viral protein in infected ST cells demonstrated by bright cytoplasmic immunofluorescent staining (Fig. 7a) and within intestinal enterocytes stained immunohistochemically (Fig. 7c).

## Discussion

As a recently identified pathogen, the impact of PDCoV on the swine industry is not yet fully understood. Field observations and recent research studies have suggested that the virus can cause substantial morbidity and mortality in nursing piglets [5–7, 17]. Specific antibody-based reagents and serological tests are essential for the further study and control of PDCoV, as well as the differentiation of PDCoV infection from other related coronaviruses such as PEDV or TGEV.

Therefore, the primary objective of this study was to develop an initial generation of antibody-based diagnostic reagents and serological assays for the further study of PDCoV, including NP-based indirect ELISA and FMIA tests that were developed and validated for diagnostic applications. Specific antibody-based reagents were also not yet available for PDCoV so monoclonal antibodies against selected PDCoV structural proteins were developed. The tools developed during the course of this study can be applied to many ongoing and future studies to better understand and control PDCoV.

The new monoclonal antibody reagents described here should be of substantial value in the detection of PDCoV antigen in a variety of applications including: early verification of virus isolation attempts and virus titrations; immunohistochemistry staining of fixed tissues; development of neutralization assays; fluorescent antibody staining of fresh tissues; development of field-based antigen capture assays such as lateral flow devices; and ELISA applications (competitive ELISA and antigen capture). Through extensive testing via ELISA (over 364 samples) and Western blot analysis, we were not able to demonstrate any cross-reactivity with other major swine coronaviruses including PEDV, PRCV and TGEV. However, since many described deltacoronaviruses of other species have not yet been adapted to cell culture replication or fully characterized, we do not yet know if these reagents may cross-react with other members of the genus.

Several new serological assays for detection of antibody responses to PDCoV were developed during the course of this study. The ELISA and FMIA tests were based on a recombinant nucleoprotein antigen since this protein is highly conserved among PDCoV isolates. In addition, the NP is known to be the most abundant viral protein within host cytoplasmic compartments [18]. The highly immunoreactive PDCoV-NP interacts with itself to form non-covalently linked oligomers and associates with the viral genome to serve as the architectural basis of the ribonucleoprotein complexes during virus assembly [19]. These reasons provided the rationale for its utility as a target antigen for the serodiagnosis of PDCoV in indirect ELISA and FMIA platforms.

Antibody responses to the nucleoprotein of coronaviruses are very robust and have been reported to appear as soon as 7–9 days post infection. We originally hypothesized that antibody reactivity to PDCoV may be conformationally dependent so we designed an experiment to determine whether NP specific immunodominant epitopes tend to be present in greater abundance on conformationally or linearly expressed antigen. A refolded version of the NP was used as an antigen in both assays as it was shown to impart a higher degree of immunoreactivity than its unfolded, linear counterpart. This phenomenon was also observed by Johnson et al. [20] whereby the authors compared reactivity differences among single point serum titrations to provide a surrogate measure of antibody titer. They showed that the enhancement of immunoreactivity of PRRSV-N and nsp1 was completely dependent on refolding, and the reactivity of nsp2P was enhanced by twofold. Furthermore we confirmed their observations that there is a loss of immunoreactivity when the linear protein (solubilized in 8 M urea) was dialyzed in PBS prior to coating on microtiter plates or used for coupling to FMIA microspheres. This may indicate that using an antigen in its more native conformational state may present a higher number of immunoreactive epitopes that are able to capture a larger percentage of PDCoV-NP antibodies. Therefore, the production of a well purified, refolded recombinant protein maintained in a near-native conformation was required for the production of an efficacious assay.

Both assays provide the capability of high-throughput testing with reasonable DSe and DSp. ROC analysis was performed for both assays demonstrating DSe of greater than 95 %. The FMIA demonstrated good DSp of 98.1 % while the ELISA showed a slightly lower DSp of 96.2 %. Inter-rater (kappa) agreement, a statistical measure of test agreement demonstrated a significant level of agreement among the IFA, ELISA and FMIA. Furthermore, each of the antibody-capture assays was validated using a large number of well characterized serum samples (*n* = 940) based upon the suggested 5-stage validation methods of Jacobson, which is supported by the office International des Epizooties [21].

Although preliminary DSe and DSp determinations for the first generation serological assays described here were slightly less than ideal, these new assays should provide valuable tools for assessment of PDCoV exposure on a herd level. One explanation for the approximately 95–96 % DSe values determined to date may be related to selection of the presumed positive field populations used for test validation. In this case, the initial stages of validation relied on characterizing the assay using known serum samples from experimentally infected pigs. Because this was the only sera of known serostatus we had at our disposal, we believe that the resulting initial ROC characteristics were of sufficient value to begin testing samples from other sources believed to not have been exposed to PDCoV and from field sources known to have seroconverted to PDCoV within a specific time frame. By following the methodology outlined by Jacobson [21] in which he recommends the inclusion of these initial ROC cut-off values from experimentally infected pigs, we demonstrated similar assay characteristics on chosen field samples which substantiated our final cut-off value of the assay. These sample sets were collected at approximately 3 weeks after initial diagnosis of PDCoV by PCR. It is possible that PDCoV may not move through a herd at the very rapid rate seen with PEDV. Therefore, some animals in some herds may not have been infected until a week or more after initial detection in the population, resulting in delayed seroconversion in a percentage of the presumed positive population. Likewise, since the initial origination and distribution of PDCoV in the U.S. is not fully understood at this time, it is possible that a small percentage of our presumed seronegative population may have been subject to prior exposure. Many of the samples in our presumed negative population were archived samples collected prior to 2009. However, some originated from recent field submissions from high health, biosecure herds with no clinical or PCR evidence of prior PDCoV or PEDV exposure. The observed DSp values of approximately 98 % for FMIA and 96 % for indirect ELISA are within the expected range for first generation assays using these test formats. Apparent false positive reactions could also be due to an epitope on the expressed antigen having commonality with another low prevalence infectious agent or due to low levels of residual *E. coli* protein contaminants in the purified antigen preparations.

Recently, Thachil et al. [8], developed an indirect anti-PDCoV IgG enzyme-linked immunosorbent assay based on the putative S1 portion of the spike protein and evaluated it using a total of 968 tested serum samples. Although it is a reasonably sensitive (91 %) and specific (95 %) test, there is room for other ELISAs utilizing other target antigens, such as the NP of PDCoV, to serve as primary serological surveillance or confirmatory assays. As noted in Thachil’s research, serum samples collected in 2010 were found positive for PDCoV antibody by their ELISA, but not those collected in 2011 and 2012. This was controversial because PDCoV was not thought to be circulating in North America in pigs before its identification in late 2013 [9]. It will be very beneficial to have confirmatory assays to validate this finding. Additional screening of archived historical swine serum samples using multiple serological assays may provide further insight into the origin and epidemiology of PDCoV in North America.

## Conclusions

The monoclonal antibody reagents developed here provide important research and diagnostic tools for the industry. They are valuable for fluorescence and immunohistochemical staining methods associated with diagnostic and pathogenesis studies. The serological assays allow the detection of antibodies developed in response to PDCoV infection. The PDCoV indirect ELISA and FMIA will allow high-throughput screening of swine serum samples. These tests should be adequately optimized and validated for sero-surveillance on a herd level, but further improvement is needed for full confidence on an individual animal basis. The IFA or other tests may be required for confirmation of individual unexpected results. Work is ongoing to further validate these assays and to adapt them to different sample matrices such as milk or oral fluid samples. Since lactogenic immunity is likely critical for protection of nursing piglets, these assays will be modified for detection of IgA as well.

## Abbreviations

ACB, Antigen coating buffer; ADRDL, Animal Disease Research and Diagnostic Laboratory; CAPS, Cyclohexylamino-1-propanesulfonic acid; CPE, Cytopathic effect; CV, Coefficient of variation; DPI, Days post infection; DSe, Diagnostic sensitivity; DSp, Diagnostic specificity; ELISA, Enzyme linked immunosorbent assay; FBS, Fetal bovine serum; FITC, Fluorescein isothiocyanate; FMIA, Fluorescent microsphere immunoassay; HRP, Horseradish peroxidase; IACUC, Institutional Animal Care and Use Committee; IFA, Indirect fluorescent antibody; mAB, Monoclonal antibody; MEM, Eagle’s minimum essential medium; MFI, Mean fluorescent intensity; NP, Nucleoprotein; NPB, National Pork Board; OD, Optical density; P/N, Positive to negative; PBS, Phosphate buffered saline; PBST, PBS plus 0.05 % tween 20; PCR, Polymerase chain reaction; PDCoV, Porcine deltacoronavirus; PEDV, Porcine epidemic diarrhea virus; PRCV, Porcine respiratory coronavirus; ROC, Receiver operator characteristic; RT-PCR, Reverse transcriptase polymerase chain reaction; S/P, Sample to positive; SDS-PAGE, Sodium dodecyl sulfate-polyacrylamide gel electrophoresis; SDSU, South Dakota State University; SMD, Sample milk diluent; ST, Swine testicle; TGEV, Transmissible gastroenteritis virus; TMB, 3,3′,5,5′-tetramethylbenzidine; TPCK, L-1-Tosylamide-2-phenylethyl chloromethyl ketone; UN-L, University of Nebraska-Lincoln; YT, Yeast extract tryptone
